# Consensus machine learning identifies cell death gene signature for carotid artery stenosis diagnosis

**DOI:** 10.1016/j.isci.2025.114397

**Published:** 2025-12-13

**Authors:** Chunguang Guo, Kun Fang, Gaopo Cai, Yi Liu, Weichang Zhang, Linfeng Zhang, Ziting Wu, Mingyao Luo, Chang Shu

**Affiliations:** 1 State Key Laboratory of Cardiovascular Disease, Center of Vascular Surgery, Fuwai Hospital, National Center for Cardiovascular Disease, Chinese Academy of Medical Sciences and Peking Union Medical Colege, Beijing 100037, China; 2 Department of Endovascular Surgery, The First Affiliated Hospital of Zhengzhou University, Zhengzhou, Henan 450052, China; 3 Department of Vascular Surgery, The Second Xiangya Hospital of Central South University, Changsha 410008, China

**Keywords:** health sciences

## Abstract

Carotid artery stenosis (CAS) is a major contributor to ischemic stroke, and molecular tools for its early detection remain limited. To address this need, we integrated one in-house RNA-seq cohort with eight public datasets comprising 696 samples, together with proteomic profiling, RT-qPCR, single-cell sequencing, and FYCO1 silencing experiments. From 1,258 curated cell death-related genes, candidates were filtered by logistic regression across cohorts, and ten machine learning algorithms were combined into 105 model configurations to derive a consensus diagnostic classifier. Fourteen genes showed consistent associations with CAS, and the machine learning-derived diagnostic signature (MLDS), consisting of IRF1, FYCO1, and FDFT1, demonstrated the highest cross-cohort performance. FYCO1 downregulation was validated in plaques and blood and supported by single-cell analysis, while functional assays indicated impaired autophagic flux and heightened inflammatory signaling. These findings highlight MLDS as a robust molecular tool that may enhance the precision diagnosis of CAS.

## Highlights

• Nine cohorts (696 samples) systematically integrated for CAS biomarker discovery
• 105 ML model configurations yielded a consensus three-gene diagnostic signature
• Transcriptomic, proteomic, single-cell, and functional assays validated FYCO1
• MLDS offers a non-invasive tool for early CAS detection and precision medicine

## Graphical abstract

**Figure:**
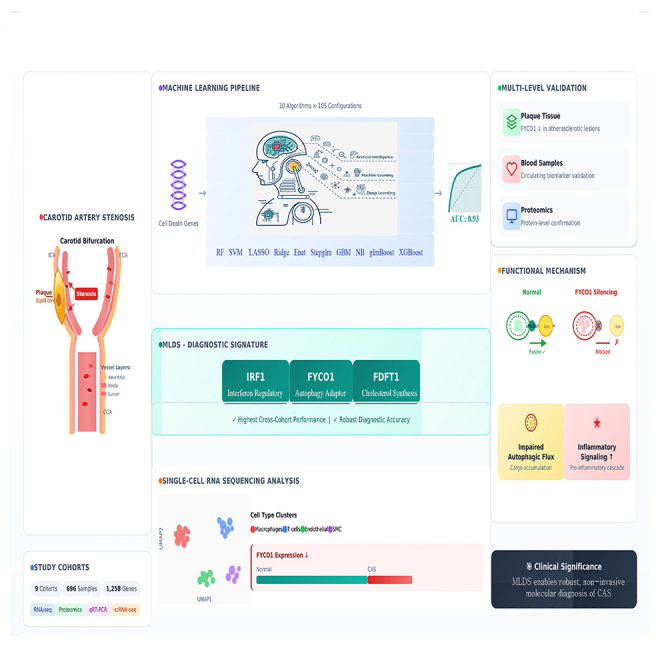

## Introduction

Carotid artery stenosis (CAS) is a major contributor to ischemic stroke, which remains the second leading cause of death worldwide.^1^ Ischemic stroke accounts for approximately 85% of all strokes,^2^ and its pathogenesis is largely attributed to insufficient cerebral perfusion and brain-tissue necrosis resulting from CAS, arterial occlusion, or plaque rupture with subsequent embolization. According to global epidemiological data, the prevalence of carotid plaques among individuals aged 30–79 years reached nearly 20% in 2020,^3^ underscoring their substantial health burden. Currently, the clinical diagnosis of CAS primarily relies on imaging techniques, including ultrasound, computed tomography angiography, and digital subtraction angiography.^4^^,^^5^ While these methods provide important morphological information, they are invasive or resource-intensive and fail to reflect the underlying molecular mechanisms of plaque development. Despite decades of research, the complexity and heterogeneity of carotid plaques mean that their pathophysiological mechanisms remain incompletely understood,^6^ and reliable molecular biomarkers for early diagnosis and targeted therapy are still lacking.

Growing evidence indicates that cell death-related processes play central roles in the initiation and progression of atherosclerosis.^7^^,^^8^ Dysregulated programmed cell death, such as apoptosis, pyroptosis, ferroptosis, and autophagy, contributes to plaque formation, inflammatory cell infiltration, and fibrous cap destabilization, thereby promoting ischemic events.^9^^,^^10^ Carotid plaques represent a complex and heterogeneous microenvironment in which vascular smooth muscle cells, macrophages, endothelial cells, and immune subsets interact through diverse death and survival pathways.^10^^,^^11^ This complexity drives disease progression but also complicates the identification of robust and reproducible biomarkers. In parallel, the advent of high-throughput sequencing and multi-omics profiling has generated extensive transcriptomic, proteomic, and single-cell datasets that enable the in-depth exploration of CAS biology.^12^^,^^13^^,^^14^ However, conventional analytic methods often struggle to integrate heterogeneous data sources or detect subtle but clinically relevant molecular signatures. Machine-learning approaches,^15^^,^^16^^,^^17^ particularly when applied in consensus frameworks, provide powerful tools to leverage large-scale data, reduce cohort-specific bias, and identify reproducible diagnostic signatures with improved generalizability.

To overcome current limitations, we integrated nine independent transcriptomic cohorts, together with proteomic and single-cell sequencing datasets, to investigate the diagnostic value of cell death-associated genes in CAS. By employing a consensus machine learning framework comprising 105 algorithmic combinations, we constructed a robust machine learning-derived diagnostic signature (MLDS) that demonstrated high diagnostic accuracy across multiple cohorts. Among the identified genes, FYCO1 emerged as a core biomarker, showing consistent downregulation in plaques and peripheral blood, and exhibiting close associations with immune infiltration and inflammatory responses. Therefore, the objectives of this study were 3-fold: (i) to construct and validate a consensus machine learning-based diagnostic signature for CAS; (ii) to explore the biological implications of MLDS genes through integrative proteomic and single-cell analyses; and (iii) to identify and characterize FYCO1 as a potential diagnostic biomarker linking immune-inflammatory regulation with CAS pathology. Collectively, our findings provide new insights into the molecular underpinnings of CAS and propose FYCO1 as a promising target for precision diagnosis and intervention.

## Results

### Integrative construction of a consensus signature

**Figure 1:**
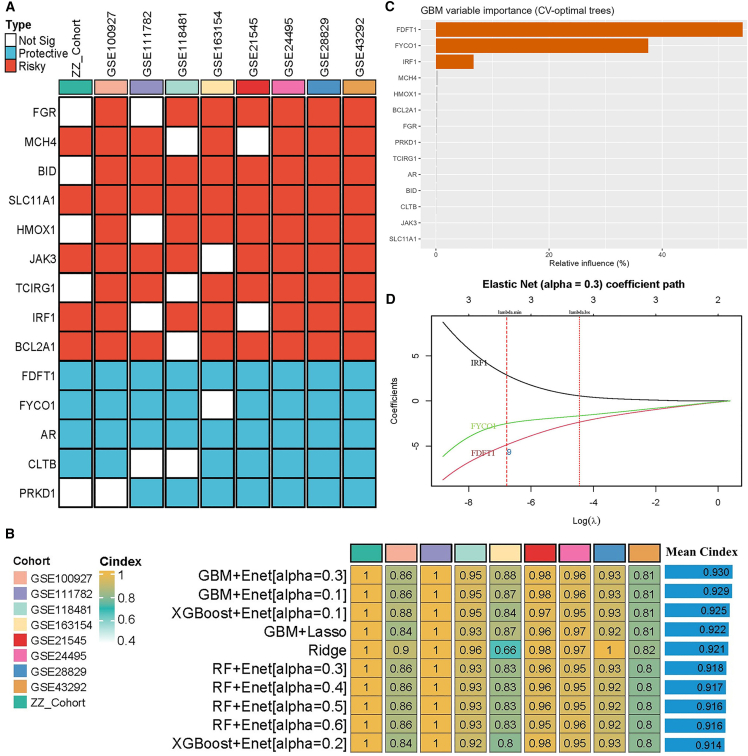
Construction of a machine learning-derived diagnostic signature in CAS (A) Identification of 14 consensus diagnostic-related genes (CDRGs) from nine cohorts. (B) Comparison of top ten machine learning models across training and validation cohorts. (C) GBM analysis highlighted FDFT1, FYCO1, and IRF1 as top predictors. (D) Elastic net regression identified a three-gene model (IRF1, FYCO1, and FDFT1), which was defined as the final MLDS.

The overall study workflow is illustrated in Figure S1. After normalization of nine independent CAS cohorts, a total of 740 cell death-associated genes were obtained by intersecting with the predefined set of 1,258 cell death-related genes. Logistic regression across cohorts, with a significance threshold (*p* < 0.05) and consistent effect directions in more than seven datasets, identified 14 consensus diagnostic-related genes (CDRGs) for further analysis (Figure 1A). To establish a robust diagnostic model, ten widely used machine learning algorithms were systematically combined into 105 algorithmic frameworks and trained in the ZZ cohort, followed by validation across eight external cohorts (Figures 1B and S2). We then calculated the mean C index of each model across all cohorts (Table S4), and found that the GBM + Enet (α = 0.3) model achieved the highest C index (0.930), which was selected for the construction of the MLDS. The detailed parameters and tuning procedures of the GBM + elastic net (α = 0.3) model are provided in the supplementary methods and Table S5. As shown in Figures 1C and 1D, gradient boosting machine (GBM) analysis highlighted FDFT1, FYCO1, and IRF1 as the most influential predictors, and elastic net regression ultimately identified a parsimonious three-gene model (IRF1, FYCO1, and FDFT1), which was defined as the consensus MLDS. The final MLDS score for each sample was calculated as follows: MLDS score = −4.86×FDFT1+2.87×IRF1–2.51×FYCO1. A higher MLDS score corresponds to a greater predicted probability of CAS.

### Evaluation of the MLDS model by ROC analysis

**Figure 2:**
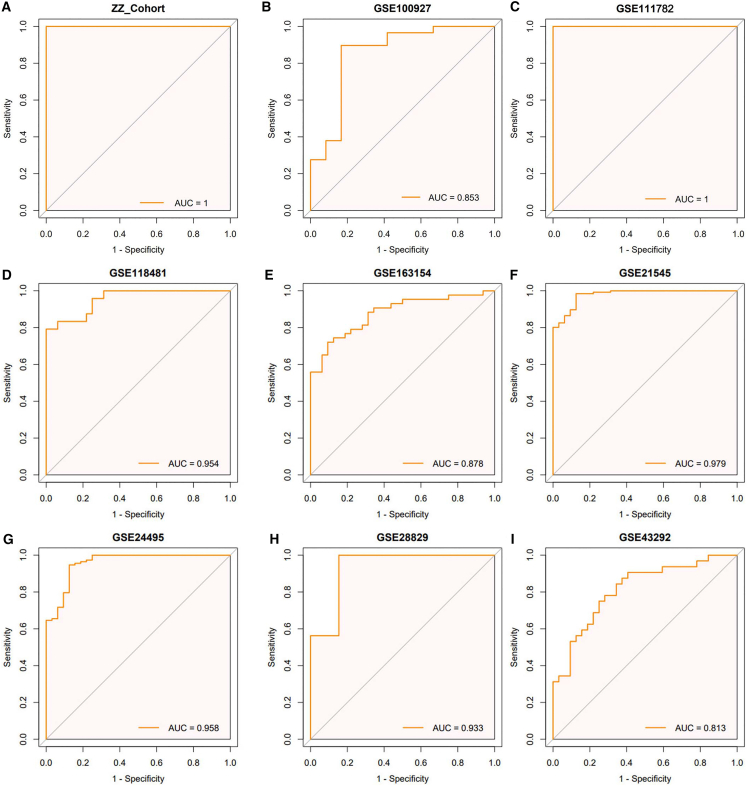
ROC curves of the MLDS in CAS Diagnostic performance of the MLDS model in the training cohort (A) and eight external validation cohorts (B–I).

To evaluate the robustness of the cohort-consistency filter, we repeated the initial logistic-regression screening using a threshold of “significant in >6 cohorts” instead of “>7 cohorts”. The resulting gene list is shown in Table S6. All three MLDS genes (IRF1, FYCO1, and FDFT1) were retained, demonstrating that the core diagnostic markers are stable with respect to the choice of threshold. Subsequently, the diagnostic performance of the three-gene MLDS was assessed in both the training and validation cohorts. Receiver operating characteristic (ROC) analysis demonstrated excellent discrimination ability, with the ZZ cohort achieving an AUC of 1.000 (Figure 2A). Consistently high predictive performance was observed in eight external cohorts (Figures 2B–2I). These results confirmed that the MLDS achieved robust diagnostic accuracy across multiple independent datasets.

### Expression validation of MLDS genes

**Figure 3:**
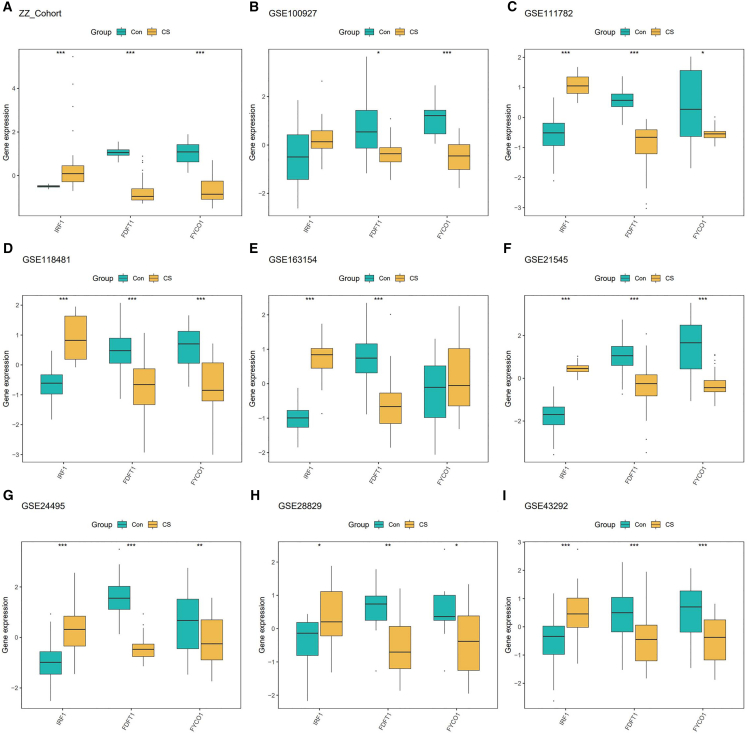
Expression validation of MLDS genes Expression levels of IRF1, FYCO1, and FDFT1 in CAS and control groups across nine cohorts (A–I). ∗*p* < 0.05, ∗∗*p* < 0.01, ∗∗∗*p* < 0.001.

We further evaluated the expression patterns of the three MLDS genes across all cohorts. IRF1, FYCO1, and FDFT1 consistently showed significant differential expression between CAS and control groups, supporting their diagnostic relevance and biological reproducibility (Figures 3A–3I). Building upon these findings, we next explored whether MLDS genes were also associated with molecular heterogeneity in CAS.

### MLDS exhibits high Specificity for CAS

To further assess whether the MLDS reflects generalized atherosclerotic burden or displays arterial-territory-specific characteristics, we applied the signature to two additional vascular stenosis cohorts: coronary artery stenosis (GSE20681) and femoral artery stenosis (GSE10092). As shown in Figure S3, none of the three MLDS genes exhibited significant differential expression in the coronary artery cohort (Figure S3A), and the overall diagnostic performance was poor (Figure S3B, AUC = 0.54). In addition, in the femoral artery stenosis cohort, the gene expression changes showed trends consistent with those observed in CAS (Figure S3C), and the MLDS achieved a moderate diagnostic performance, with an AUC of 0.75 that was still lower than in CAS (Figure S3D).

### Consensus clustering and identification of subtype-specific genes

**Figure 4:**
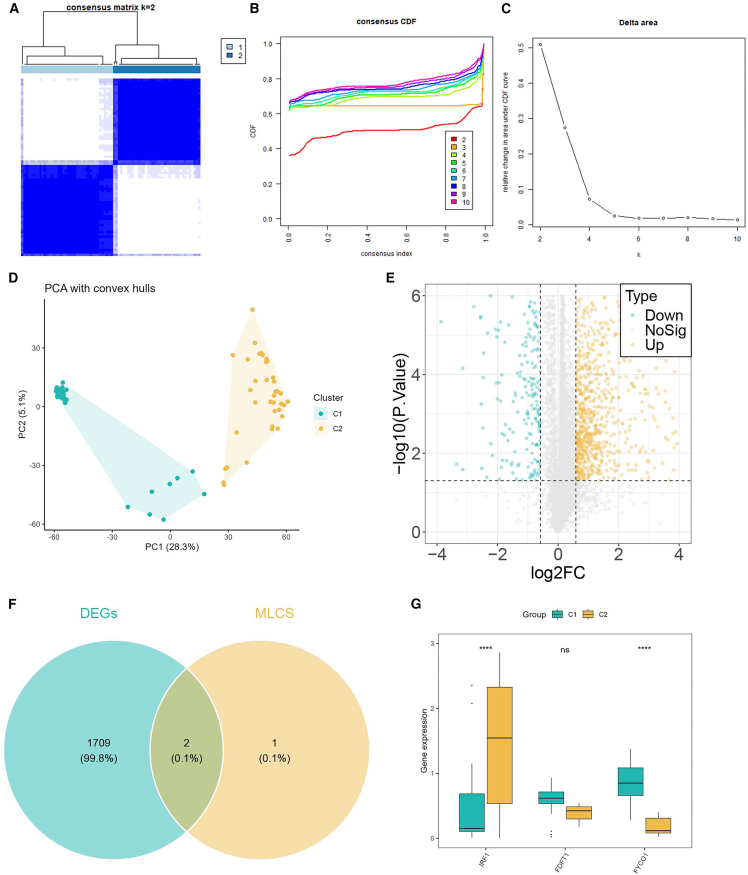
Consensus clustering and identification of subtype-specific genes (A–C) Consensus clustering defined two subtypes (C1 and C2). (D) PCA confirmed clear separation of clusters. (E) Differential expression analysis between C1 and C2. (F) Venn diagram showing overlap between DEGs and MLDS genes. (G) Expression of MLDS genes in the two subtypes. ns *p* > 0.05, ∗∗∗∗*p* < 0.0001.

Initially, consensus clustering of the ZZ cohort divided patients into two stable molecular subtypes (C1 and C2), as supported by the consensus matrix, cumulative distribution function curve, and delta area plot (Figures 4A–4C). Subsequently, principal-component analysis further confirmed a clear separation between the two clusters (Figure 4D). To further explore subtype specific alterations, differential expression analysis was performed between C1 and C2, which identified 805 downregulated genes and 906 upregulated genes (Figure 4E). By intersecting differentially expressed genes (DEGs) with MLDS genes, two overlapping genes were identified (Figure 4F). Finally, expression analysis showed that IRF1 and FYCO1 were significantly different between the two clusters (Figure 4G). These findings indicate that MLDS genes, particularly IRF1 and FYCO1, contribute to the molecular heterogeneity of CAS subtypes. Additionally, we found that patients classified into the C2 subtype exhibited significantly higher MLDS scores compared with the C1 subtype (Figure S3E).

### FYCO1 is significantly downregulated in carotid plaques

**Figure 5:**
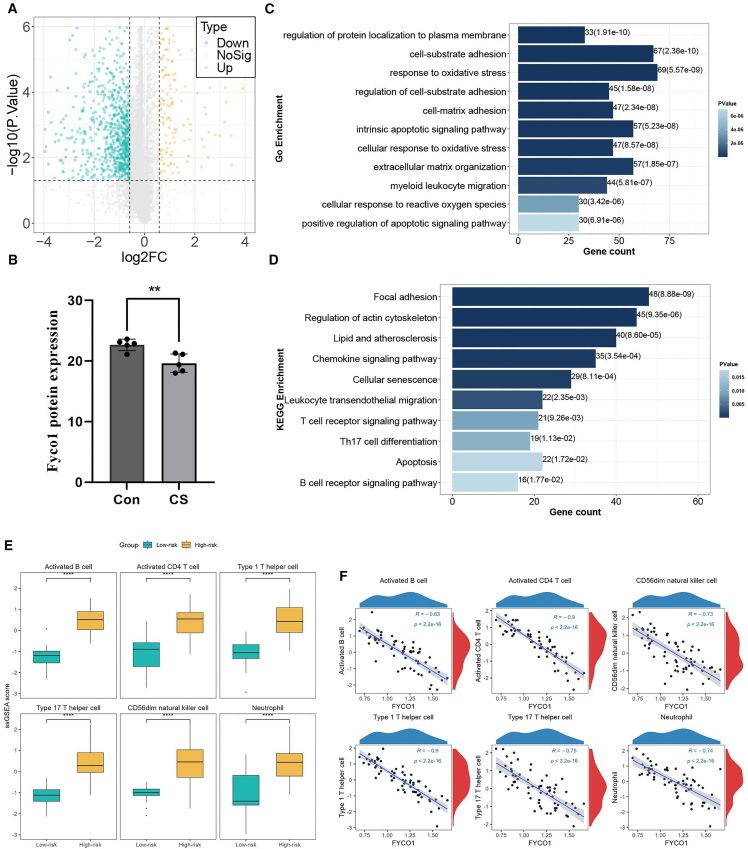
Functional analysis and immune correlations of FYCO1 in CAS (A) Volcano plot of DEGs between high- and low-risk groups. (B) Protein expression of FYCO1 in plaques and adjacent normal tissues. (C and D) GO and KEGG enrichment of DEGs in high-risk patients. (E) Immune infiltration differences between high- and low-risk groups. (F) Negative correlations between FYCO1 expression and immune cell abundance. ∗∗*p* < 0.01, ∗∗∗∗*p* < 0.0001.

Based on the MLDS model, a risk score was calculated for each patient, and individuals were stratified into high- and low-risk groups according to the optimal cutoff value. Volcano plot (Figure 5A) analysis revealed DEGs between the two groups, among which the MLDS genes all showed significant differences. To further validate these findings at the protein level, we performed proteomic analysis of carotid plaques and adjacent normal vascular tissues. Notably, only FYCO1 exhibited a significant difference between plaque and normal tissues, confirming its specific downregulation in carotid atherosclerosis (Figure 5B).

### Enhanced immune and inflammatory infiltration in carotid plaques

Gene set enrichment analysis (GSEA) was performed to explore the biological differences between the MLDS-defined risk groups. High-risk patients were predominantly enriched in pathways associated with immune activation and inflammation, including focal adhesion, leukocyte *trans*-endothelial migration, chemokine signaling, T cell receptor signaling, and Th17 cell differentiation (Figures 5C, 5D, S4C, and S4D). In contrast, the low-risk group showed enrichment in processes related to cellular metabolism and structural maintenance, such as protein transport, synaptic signaling, DNA replication, and axon guidance (Figures S4A and S4B). These findings suggest that the MLDS risk stratification reflects distinct biological programs, with immune-inflammatory activation characterizing high-risk patients and metabolic/structural functions predominating in the low-risk group.

### Negative correlations between FYCO1 and immune cell infiltration

To further investigate the relationship between MLDS genes and immune status, we examined the association between FYCO1 expression and immune infiltration. In the MLDS-defined risk groups, the high-risk group exhibited significantly higher infiltration of six major immune cell subsets, including activated B cells, activated CD4 T cells, type 1 and type 17 T helper cells, CD56dim natural killer cells, and neutrophils, compared with the low-risk group (Figure 5E). Correlation analysis confirmed that FYCO1 expression was negatively correlated with the infiltration levels of these immune cells (Figure 5F). Consistently, similar results were observed in the consensus clustering subtypes. Patients in the C2 subtype showed significantly higher immune infiltration than those in the C1 subtype (Figure S4E). Moreover, FYCO1 expression displayed robust negative correlations with immune cell abundance across subtypes (Figure S4F).

### Diagnostic performance and expression validation of FYCO1

**Figure 6:**
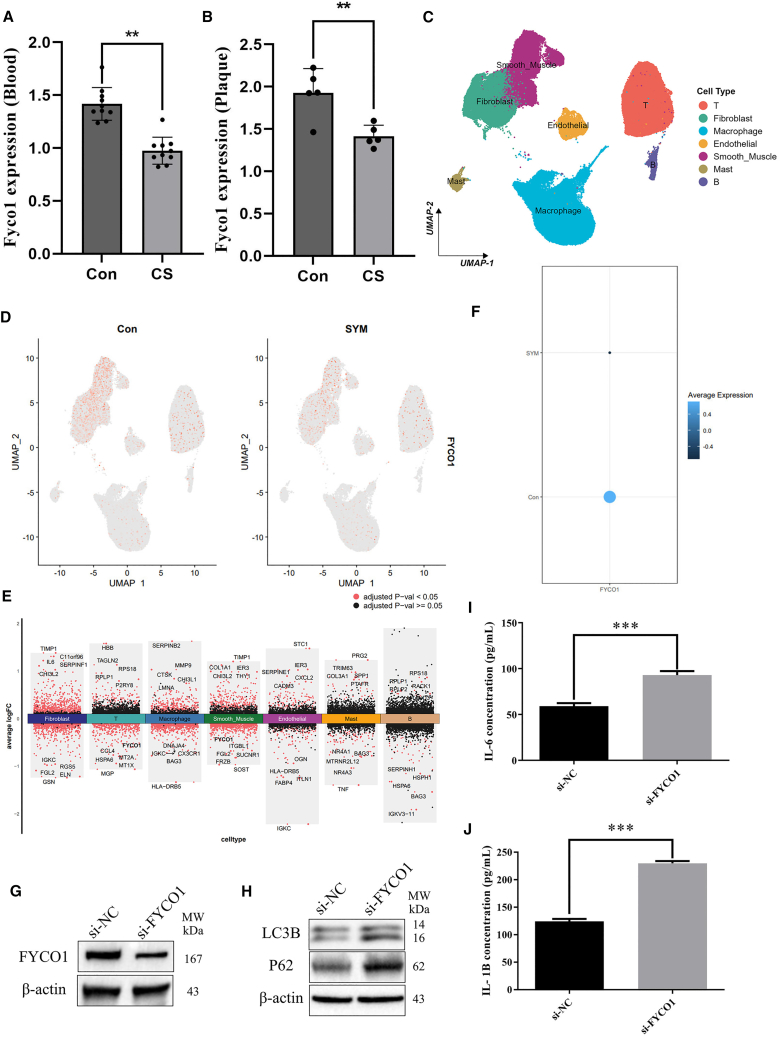
Validation of FYCO1 expression in blood, plaque, and single-cell data (A and B) RT-qPCR showed reduced FYCO1 expression in blood and plaques compared with controls. (C) UMAP visualization of major cell types. (D) Distribution of FYCO1 expression in control and symptomatic samples. (E) Differentially expressed genes across cell types. (F) Average FYCO1 expression showing marked downregulation in plaques. (G) Western blot confirming effective FYCO1 knockdown in macrophages. (H) FYCO1 silencing induced autophagic flux impairment. (I and J) ELISA showing significantly elevated IL-6 and IL-1β secretion in FYCO1-deficient macrophages. ∗∗*p* < 0.01, ∗∗∗*p* < 0.001.

ROC analysis demonstrated that FYCO1 had strong diagnostic value in the training cohort (AUC = 0.988) and maintained moderate to high performance in eight external cohorts, with AUCs ranging from 0.601 to 0.951 (Figures S5A–S5I). To further validate these findings, RT-qPCR analysis showed that FYCO1 expression was significantly reduced in the blood of CAS patients compared with healthy controls (Figure 6A) and was also markedly lower in carotid plaques relative to adjacent normal vascular tissues (Figure 6B). Furthermore, we have discovered that FYCO1 still exhibits strong diagnostic efficacy in peripheral blood samples (Figure S3F). These results confirm that FYCO1 is consistently downregulated in CAS at both the transcriptional and protein levels.

### Single-cell validation of FYCO1 expression

To further validate the expression of FYCO1 at the single-cell level, we analyzed carotid plaque and control vascular tissues using single-cell RNA sequencing. Quality control and batch effect correction ensured reliable integration of samples (Figures S6A–S6E). Cell clustering identified major cell populations, including T cells, macrophages, smooth muscle cells, fibroblasts, endothelial cells, mast cells, and B cells (Figures 6C and S6F). Expression analysis revealed that FYCO1 was markedly downregulated in CAS plaques compared with controls (Figures 6D and 6F). Importantly, compared to normal vascular tissue, the differential expression analysis across cell subsets revealed that in carotid plaque tissue, FYCO1 was significantly reduced in T cells (Figure 6E). These results provide strong single-cell evidence that FYCO1 is suppressed in key cell types driving carotid atherosclerosis.

### FYCO1 knockdown impairs autophagic flux and enhances inflammatory cytokine production

FYCO1 expression was markedly reduced following siRNA transfection, confirming effective knockdown (Figure 6G). FYCO1 silencing resulted in autophagic flux impairment, as reflected by the accumulation of LC3B and p62 proteins (Figure 6H). Moreover, inflammatory responses were significantly amplified in the FYCO1-deficient cells, with both IL-6 (Figure 6I) and IL-1β (Figure 6J) levels markedly elevated in the culture supernatants compared with the si-NC group.

## Discussion

CAS remains a major contributor to ischemic stroke,^1^^,^^3^ but reliable molecular biomarkers for early diagnosis and risk prediction are still lacking. In this study, we constructed and validated a robust MLDS for CAS by integrating nine transcriptomic datasets and validating the results with proteomic and single-cell sequencing data. The MLDS, consisting of three genes, showed excellent diagnostic performance across multiple independent cohorts. Notably, FYCO1 emerged as a particularly promising biomarker, consistently downregulated in carotid plaques, adjacent normal vascular tissues, and peripheral blood of CAS patients. At the single-cell level, FYCO1 expression was significantly reduced in immune cells, suggesting its close link to immune-inflammatory dysregulation and plaque instability.

Prior efforts to improve CAS diagnosis have largely centered on imaging markers or circulating proteins, yet recent reviews conclude that reliable blood biomarkers for carotid plaque vulnerability remain unestablished, and most candidates suffer from limited reproducibility across cohort.^18^ Methodologically, many genomics-based signatures have been derived in single datasets or with a single algorithm, with sparse external validation.^19^^,^^20^^,^^21^ Compared to above researches, our findings provide several important insights. First, the use of a consensus machine-learning framework integrating 105 algorithmic models across multiple datasets enabled the identification of reproducible biomarkers with high generalizability. Unlike previous studies that relied on a single dataset or algorithm,^22^ this approach minimized cohort-specific bias and avoided overfitting, thereby increasing robustness. Second, multi-level validation at transcriptomic, proteomic, and single-cell levels provided convergent evidence, ensuring that the identified markers are not only statistically significant but also biologically relevant.

Compared with conventional imaging modalities, such as duplex ultrasound, and digital subtraction angiography, which provide morphological but not molecular information,^5^^,^^23^^,^^24^ MLDS offers a minimally invasive and biologically informed tool for CAS diagnosis. Unlike earlier molecular signatures^25^^,^^26^ often derived from a single cohort or algorithm with limited external validation, our consensus framework integrated nine cohorts and 105 algorithmic combinations, thereby enhancing reproducibility and generalizability. Importantly, MLDS integrates three genes (IRF1, FDFT1, and FYCO1), which collectively represent immune regulation, lipid metabolism, and autophagy, capturing the multidimensional nature of plaque biology. These features highlight the clinical potential of MLDS as a complement to imaging, enabling earlier detection, risk stratification, and longitudinal monitoring of CAS patients. Among the three MLDS genes, FYCO1 deserves particular attention. Its consistent downregulation across transcriptomic, proteomic, and single-cell levels suggests a mechanistic role in plaque progression, linking impaired autophagy to immune-inflammatory dysregulation and vascular instability.

Notably, the downregulation of FYCO1 across multiple biological levels may have important mechanistic implications. FYCO1 encodes an autophagy adapter protein that mediates the transport of autophagosomes along microtubules through interactions with LC3 and Rab7.^27^ Reduced FYCO1 expression could impair autophagosome clearance, leading to the accumulation of damaged organelles and increased oxidative stress.^28^ Such autophagy dysfunction has been implicated in multiple pro-atherogenic processes. These mechanistic implications are supported by our findings showing strong inverse correlations between FYCO1 expression and multiple immune-infiltrating cell populations in carotid plaques. Defective autophagy can amplify inflammatory signaling through NLRP3 inflammasome activation, impaired efferocytosis, and the release of damage-associated molecular patterns, all of which drive vascular inflammation and plaque progression. Our external validation experiments further strengthen this interpretation: FYCO1 knockdown reduced LC3B, increased P62 accumulation, and markedly elevated IL-6 and IL-1β secretion, demonstrating that diminished FYCO1 directly disrupts autophagy and enhances inflammatory cytokine production. Previous studies have linked FYCO1 to multiple biological contexts. Fassi et al.^29^ reported that FYCO1 peptide analogs act as autophagy inhibitors and function as co-adjuvants in taxane chemotherapy for prostate cancer. Chen et al. demonstrated that FYCO1 regulates autophagy and senescence via the PAK1/p21 pathway in cataract.^30^ Other investigations further showed that FYCO1 modulates cardiomyocyte autophagy and prevents pressure overload-induced heart failure *in vivo*.^31^ However, research on FYCO1 in CAS remains absent, and our study provides the first evidence implicating its downregulation and immune-inflammatory relevance in atherosclerotic plaques. Moreover, we observed reduced FYCO1 levels in the peripheral blood of CAS patients, suggesting its potential utility as a minimally invasive biomarker for disease screening and monitoring, which is highly attractive in clinical practice.^32^^,^^33^

While retrospective studies enable efficient leveraging of existing resources and facilitate multi-cohort integration, they are inherently subject to selection bias. Despite these limitations, we believe that the development of MLDS represents an important step toward molecularly informed diagnosis of CAS. By integrating multi-cohort transcriptomic data with proteomic and single-cell validation, our study provides a reproducible and biologically grounded framework for biomarker discovery. In particular, the identification of FYCO1 as a consistently downregulated gene not only adds novel insight into plaque biology but also suggests a promising target for future translational research. Collectively, these findings may help bridge the gap between conventional imaging and molecular diagnostics, ultimately facilitating earlier detection, improved risk stratification, and precision management of patients with CAS.

In this study, we developed and validated a robust MLDS for CAS using nine transcriptomic cohorts, supported by proteomic and single-cell RNA sequencing data. By applying a consensus machine-learning framework integrating 105 models, we identified a parsimonious three-gene signature (IRF1, FDFT1, and FYCO1) with consistently high diagnostic accuracy across independent datasets. Among these, FYCO1 emerged as a promising biomarker, showing downregulation in plaques, adjacent tissues, blood, and immune cell subsets, linking autophagy dysregulation to immune-inflammatory activity and plaque vulnerability. Our findings provide new insights into CAS biology and highlight the potential of MLDS as a complementary diagnostic tool to imaging, with applications in early detection, risk stratification, and monitoring. Despite the retrospective design and limited sample sizes, this study demonstrates the value of combining multi-omics validation with consensus machine learning and provides a strong rationale for translational and mechanistic investigations of FYCO1 in CAS.

### Limitations of the study

However, our study has limitations. First, although we analyzed multiple datasets, some cohorts were relatively small, which may affect statistical robustness. Second, although we applied the ComBat algorithm to correct for batch effects across different platforms and cohorts, residual technical variation cannot be entirely excluded, particularly when integrating RNA-sequencing and microarray data generated with diverse protocols. Third, immune cell infiltration levels were estimated using computational algorithms (ssGSEA) rather than direct flow cytometry or immunohistochemical quantification, which may introduce bias due to the inherent assumptions of deconvolution methods. Fourth, it is important to acknowledge that all datasets used in this study, including both public GEO cohorts and our in-house ZZ cohort, are retrospective in nature. While retrospective studies enable efficient leveraging of existing resources and facilitate multi-cohort integration, they are inherently subject to selection bias. Fifth, the proteomic validation in this study, while highly consistent with the transcriptomic data, was conducted in a relatively small sample set; future studies with larger cohorts are warranted to confirm these findings.

## Resource availability

### Lead contact

Requests for further information and resources should be directed to and will be fulfilled by the lead contact, Chang Shu (changshu@vip.126.com).

### Materials availability

This study did not generate new unique reagents.

### Data and code availability

• All data reported in this article will be shared by the lead contact upon request.
• Data and code have been deposited at GitHub and are publicly available as of the date of publication. Accession numbers are listed in the key resources table.
• Any additional information required to reanalyze the data reported in this article is available from the lead contact upon request.

## Acknowledgments

This study was funded by the Capital’s Funds for Health Improvement and Research (no. 2024-1-4031), Central China Subcenter of National Center for Cardiovascular Diseasese (no. 2023-FZX12), and Chinese National High Level Hospital Clinical Research Funding (no. 2024-GSP-TJ-3). We acknowledge Sogen Biotechnology, Zhengzhou, Henan Province, and the SolvingLab team for their assistance and support.

## Author contributions

C.G. and C.S. designed this work. C.G., K.F., G.C., Y.L., W.Z., L.Z., Z.W., M.L., and C.S. edited and revised the manuscript. All authors approved this manuscript.

## Declaration of interests

The authors declare no competing interests.

## STAR★Methods

### Key resources table

**Table:** 

REAGENT or RESOURCE	SOURCE	IDENTIFIER
**Antibodies**		

FYCO1 Polyclonal antibody	Proteintech	RRID: AB_3085821
LC3B (D11) Rabbit Monoclonal Antibody	Cell Signaling Technology	RRID: AB_2137707
SQSTM1/p62 Antibody	Cell Signaling Technology	RRID: AB_10624872
Beta Actin Monoclonal antibody	Proteintech	RRID: AB_2687938
HRP-conjugated Goat Anti-Rabbit IgG(H + L)	Proteintech	RRID: AB_2722564
Multi-rAb™ HRP-Goat Anti-Mouse Recombinant Secondary Antibody (H + L)	Proteintech	RRID: AB_3068333

**Chemicals, peptides, and recombinant proteins**		

Human IL-1 beta ELISA Kit	Proteintech	KE00021

AuthentiKine™ Human IL-6 ELISA Kit	Proteintech	KE00139
Phorbol 12-myristate 13-acetate	Sigma	P8139-1 MG

**Experimental models: Cell lines**		

THP-1	Pricella	CM-0233

**Software and algorithms**		

SuPerMax 3200 Multimode microplate reader	Flash	http://www.shanpu2010.com/productinfo/1812317.html

Tanon5200	Tanon	https://biotanon.com/Product/ProductList?id=318&type=wholedetail
Fluorescence quantitative polymerase chain reaction (PCR) detection system	BIOER	https://www.bioer.com.cn/product/info.aspx?itemid=126&lcid=16
Data and code	GitHub	herehttps://github.com/guochunguang1/MLDS-for-CAS
Rversion4.4.2	https://www.r-project.org/	https://www.r-project.org/

**Other**		

Bulk microarray or RNA-seq and clinical data	https://www.ncbi.nlm.nih.gov/geo/query/acc.cgi?acc=GSE100927	GSE100927

Bulk microarray or RNA-seq and clinical data	https://www.ncbi.nlm.nih.gov/geo/query/acc.cgi?acc=GSE111782	GSE111782
Bulk microarray or RNA-seq and clinical data	https://www.ncbi.nlm.nih.gov/geo/query/acc.cgi?acc=GSE118481	GSE118481
Bulk microarray or RNA-seq and clinical data	https://www.ncbi.nlm.nih.gov/geo/query/acc.cgi?acc=GSE163154	GSE163154
Bulk microarray or RNA-seq and clinical data	https://www.ncbi.nlm.nih.gov/geo/query/acc.cgi?acc=GSE21545	GSE21545
Bulk microarray or RNA-seq and clinical data	https://www.ncbi.nlm.nih.gov/geo/query/acc.cgi?acc=GSE24495	GSE24495
Bulk microarray or RNA-seq and clinical data	https://www.ncbi.nlm.nih.gov/geo/query/acc.cgi?acc=GSE28829	GSE28829
Bulk microarray or RNA-seq and clinical data	https://www.ncbi.nlm.nih.gov/geo/query/acc.cgi?acc=GSE43292	GSE43292

### Experimental model and study participant details

#### Cell culture, and treatments

Human monocytic THP-1 cells (Pricella, Hubei, China) were maintained in RPMI-1640 medium (Gibco, USA) supplemented with 10% fetal bovine serum (Gibco, Melbourne, Australia) at 37 °C in a humidified incubator containing 5% CO_2_. For macrophage differentiation, THP-1 cells were seeded into 6-well plates and treated with 100 ng/mL phorbol 12-myristate 13-acetate (Merck, Shanghai, China) for 24 h, followed by a 24-h resting period in PMA-free complete medium. Differentiated THP-1-derived macrophages were divided into two groups: a negative control siRNA group (si-NC) and an FYCO1-silencing group (si-FYCO1). Transient transfection was performed using Lipofectamine RNAiMAX reagent (Thermo Fisher, Massachusetts, USA) according to the manufacturer’s protocol. Cells and culture supernatants were collected 48 h after transfection for subsequent experiments. Western blot assays were conducted to assess FYCO1 expression and autophagy-related proteins, including LC3B and P62, to evaluate alterations in autophagic activity following FYCO1 knockdown. Levels of pro-inflammatory cytokines IL-1β and IL-6 in cell culture supernatants were quantified using commercial ELISA kits (Proteintech, Hubei, China).

### Method details

#### Data collection and processing

A total of 11 datasets were integrated in this study to comprehensively investigate carotid atherosclerotic plaques. One in-house RNA sequencing cohort (ZZ-Cohort) was generated from carotid plaque specimens collected during carotid endarterectomy at the Department of Vascular Surgery, The First Affiliated Hospital of Zhengzhou University (Ethical Number: 2023- KY-1133-002). Detailed baseline information of all included patients is summarized in Table S1. In addition, eight independent transcriptomic datasets were retrieved from the Gene Expression Omnibus (GEO) databases (GSE100927, GSE111782, GSE118481, GSE163154, GSE21545, GSE24495, GSE28829, and GSE43292), and their detailed information is provided in Table S2. Furthermore, one in-house proteomic dataset (ZZ-Protein-Cohort) and one in-house single-cell RNA sequencing dataset (ZZ-SC-Cohort) of carotid plaques were included for multi-omics validation and mechanistic exploration. Among these, the ZZ-Cohort was treated as the training dataset, whereas the remaining datasets served as independent validation cohorts.

For RNA sequencing data, raw read counts were transformed into transcripts per million (TPM) and subsequently log2-transformed. For microarray datasets from GEO and ArrayExpress, raw expression values were processed using the robust multi-array average (RMA) algorithm implemented in the *Affy* package. Probe re-annotation was performed based on the GENCODE (GRCh38) reference genome, and when multiple probes corresponded to the same gene, their average value was taken as the final expression level. Batch effects across different cohorts and platforms were corrected using the *ComBat* function in the *sva* package.^34^ Prior to feature selection and model construction, all expression profiles were standardized to Z-scores within each cohort, ensuring comparability of gene expression distributions. The ZZ-Protein-Cohort was analyzed to validate the translational relevance of identified gene signatures at the protein level, while the in-house single-cell dataset was used to dissect the cellular heterogeneity of carotid plaques and to explore the cell type specific expression of candidate genes.

#### Cell death-related gene collection

To capture cell death associated molecular features, we curated 1,258 regulators involved in 12 distinct cell death modalities, including apoptosis, necroptosis, pyroptosis, ferroptosis, autophagy-dependent cell death, cuproptosis, alkaliptosis, parthanatos, oxeiptosis, entotic cell death, lysosome-dependent cell death, and neutrophil extracellular trap (NET)-related cell death. All genes could be retrieved in Table S3. These regulators were obtained from the Molecular Signatures Database (MSigDB) and previously published literature.^35^^,^^36^ They were used to characterize cell death signatures across all included cohorts and to construct predictive models.

#### Consensus machine learning-derived diagnosis signature pipeline

Based on previous studies,^37^^,^^38^^,^^39^^,^^40^ we constructed a pipeline to generate a consensus machine learning-derived diagnosis signature (MLDS) in Carotid artery stenosis (CAS) patients based on the following steps:

Step 1: Data application. Nine independent CAS cohorts (ZZ-Cohort, GSE100927, GSE111782, GSE118481, GSE163154, GSE21545, GSE24495, GSE28829, and GSE43292) with complete gene expression information were assigned to construct MLDS. ZZ-Cohort was assigned as the training dataset, while the remaining eight cohorts served as external validation datasets.

Step 2: Feature screening. To identify diagnosis-related candidate genes, logistic regression was applied in each cohort. Genes with *p* values <0.05 and consistent effect directions in more than seven cohorts were retained as consensus diagnostic-related genes (CDRGs). This strategy was adopted to minimize the influence of small-sample cohorts and enhance the robustness of gene selection.

Step 3: Consensus signature exploration. Ten widely used machine learning algorithms were employed, including least absolute shrinkage and selection operator (LASSO), random forest (RF), elastic net (Enet), ridge regression, gradient boosting machine (GBM), generalized linear model boosting (glmBoost), support vector machine (SVM), naive Bayes, eXtreme gradient boosting (XGBoost), and stepwise logistic regression (Stepglm). Based on these 10 algorithms, a total of 105 different algorithm combinations were systematically constructed to establish diagnostic prediction models. All models were trained on the ZZ-Cohort with 10-fold cross-validation. The details of algorithm parameter tuning are described in the supplementary methods.

Step 4: Identification of MLDS. The diagnostic performance of 105 models was evaluated across the eight validation cohorts. The area under the receiver operating characteristic curve (AUC) was calculated for each model in every cohort, and the model achieving the highest average AUC across validation datasets was identified as the final MLDS.

#### Consensus clustering

Consensus clustering was performed on the ZZ-Cohort using the *ConsensusClusterPlus* package^41^ in R to identify potential molecular subtypes. Patients were classified into two subtypes (C1 and C2). Principal component analysis (PCA) was used to visualize the clustering results. Differentially expressed genes (DEGs) between C1 and C2 were identified using the *limma* package, and the overlap between DEGs and MLDS genes was extracted for subsequent analysis.

#### Proteome analysis

For proteomic validation, carotid atherosclerotic plaques from five CAS patients and the corresponding adjacent normal vascular tissues were subjected to proteomic profiling. Protein extraction and enzymatic digestion were performed following standard protocols. Peptides were analyzed using an Orbitrap Fusion Lumos mass spectrometer (Thermo Fisher Scientific, USA). Raw data were processed with MaxQuant software integrated with the Andromeda search engine against the UniProt human database. Differential protein expression between plaque and adjacent normal tissues was determined, and the protein-level expression of MLDS genes was further evaluated to confirm their diagnostic relevance.

#### Enrichment analysis

A risk score for each patient was calculated based on the expression of MLDS genes. Patients in the ZZ-Cohort were then stratified into high-risk and low-risk groups according to the optimal cutoff value. Due to its sensitivity to biological processes, gene set enrichment analysis (GSEA) was often utilized to explore the enrichment status of gene lists in specific functional pathways.^42^ We performed the differential analysis of all genes by ‘limma’ R package in high- and low-risk groups and ranked genes according to log2FoldChange (log2FC). Based on the gene rank list, the underlying biological mechanism of high- and low-risk groups were deciphered by GO and KEGG terms in GSEA.

#### Immune microenvironment

The relative infiltration levels of immune cells were assessed using the single-sample gene set enrichment analysis (ssGSEA) algorithm.^43^^,^^44^ We focused on six representative immune subsets (activated B cells, activated CD4 T cells, type 1 T helper cells, type 17 T helper cells, CD56dim natural killer cells, and neutrophils) for subsequent analysis. The correlation between FYCO1 expression and the abundance of these six immune cells was further evaluated using Spearman correlation analysis. In parallel, the same approach was applied to compare immune cell infiltration differences between the C1 and C2 subtypes derived from consensus clustering.

#### Human specimens and quantitative real-time PCR

Peripheral blood samples from five patients with CAS and five healthy controls, as well as five carotid atherosclerotic plaques with their paired adjacent normal vascular tissues, were collected at the Department of Vascular Surgery, The First Affiliated Hospital of Zhengzhou University. All participants provided informed consent, and the study was approved by the institutional ethics committee. Total RNA was extracted using RNAiso Plus reagent (Takara, Japan), and RNA concentration and purity were assessed with a NanoDrop spectrophotometer (Thermo Fisher Scientific, USA). Complementary DNA was synthesized with a reverse transcription kit (Takara, Japan), and quantitative real-time PCR was performed with SYBR Green Master Mix (Applied Biosystems, USA) on a QuantStudio Real-Time PCR System (Thermo Fisher Scientific, USA). The relative expression level of FYCO1 was normalized to GAPDH, and fold changes were calculated using the 2ˆ−ΔΔCt method.

#### Single-cell RNA sequencing analysis

Carotid atherosclerotic plaque tissues from 10 patients undergoing surgery at the Department of Vascular Surgery, The First Affiliated Hospital of Zhengzhou University, were collected for single-cell RNA sequencing. Fresh samples were enzymatically dissociated into single-cell suspensions following standard protocols, and cell viability was assessed prior to library preparation. Single-cell libraries were generated using a droplet-based microfluidics platform and sequenced on the Illumina platform to obtain high-throughput transcriptomic profiles. Raw sequencing data were processed for quality control to remove low-quality cells and potential doublets. Cells with extreme gene counts, very low gene detection, or excessive mitochondrial gene content were excluded. After filtering, gene expression matrices were constructed and normalized. Dimension reduction was performed using principal component analysis (PCA), followed by clustering with a graph-based algorithm. Visualization of clusters was achieved with t-distributed stochastic neighbor embedding (t-SNE) or uniform manifold approximation and projection (UMAP). Cell-type annotation was assigned according to canonical marker genes with the assistance of the Seurat packages.^45^ Differential expression analysis was conducted across clusters. Importantly, the expression pattern of FYCO1 was specifically examined at the single-cell level, and its cell type specific distribution was evaluated to clarify which cell populations contribute to its dysregulation in carotid atherosclerosis.

#### FYCO1 knockdown and functional validation in THP-1 derived macrophages

THP-1 cells were cultured in RPMI-1640 medium supplemented with 10% fetal bovine serum and differentiated into macrophages by treatment with 100 ng/mL PMA for 24 h followed by a 24-h resting period. Differentiated cells were divided into two groups: a negative control siRNA group (si-NC) and an FYCO1 knockdown group (si-FYCO1). siRNA transfection was performed using Lipofectamine RNAiMAX according to the manufacturer’s instructions, and cells together with supernatants were collected 48 h after transfection for subsequent analyses.

Western blotting was used to examine FYCO1 expression and autophagy-related proteins, including LC3B and P62, to evaluate changes in autophagic flux following FYCO1 silencing. Levels of IL-1β and IL-6 in culture supernatants were measured using ELISA to assess inflammatory cytokine production. These assays were performed to characterize the functional consequences of FYCO1 knockdown on autophagy and inflammation in THP-1–derived macrophages.

### Quantification and statistical analysis

Statistical analyses of FYCO1 expression and related bar plots were performed using GraphPad Prism (version 9.5). All other data processing, statistical testing, and visualization were conducted with R software (version 4.4.2). Differences between two groups were assessed using the Student’s *t* test (for normally distributed data) or the Wilcoxon rank-sum test (for non-parametric data). Correlations between gene expression and immune cell infiltration were evaluated with Spearman’s correlation analysis. The diagnostic performance of MLDS and candidate genes was assessed using receiver operating characteristic (ROC) curve analysis. Unless otherwise specified, two-tailed *p* values <0.05 were considered statistically significant.

## Supplemental information

Supplementary Methods

Table S1. Basic information of patients for single-cell sequencing samples

Table S2. List and Processing of GEO Data Sets

Table S3. Gene lists for 12 cell death modalities

Table S4. C-index of all machine-learning models

Table S5. Hyperparameters of the final GBM

Table S6. Gene list from robustness analysis using a >6-cohort threshold
