# Supplementary material for: Consensus machine learning identifies cell death gene signature for carotid artery stenosis diagnosis
Source: iScience. 2025 Dec 13;29(2):114397. doi: 10.1016/j.isci.2025.114397 (PMC12874109; doi:10.1016/j.isci.2025.114397)
Supplement: Document S1. Figures S1–S6 [file mmc1.pdf]

iScience, Volume *29*

Supplemental information

Consensus machine learning identifies cell death gene signature for carotid artery stenosis diagnosis

Chunguang Guo, Kun Fang, Gaopo Cai, Yi Liu, Weichang Zhang, Linfeng Zhang, Ziting Wu, Mingyao Luo, and Chang Shu

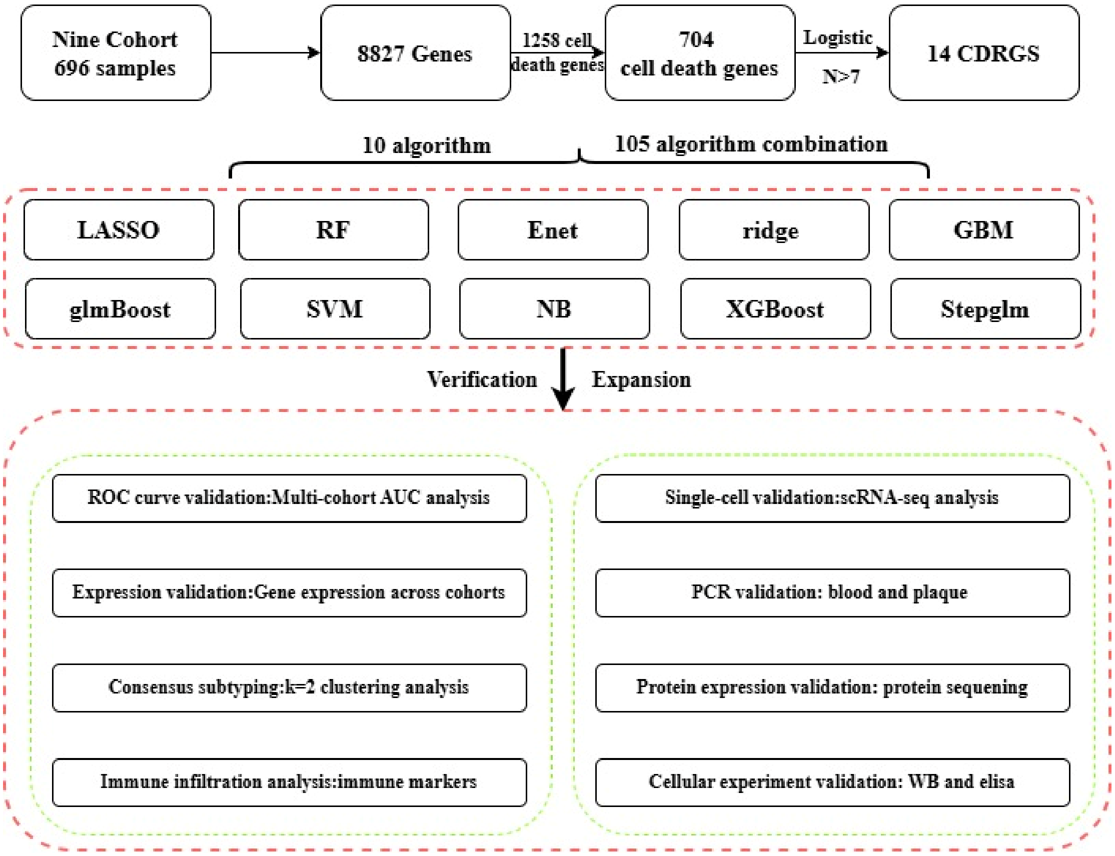

Figure S1. The overall study workflow

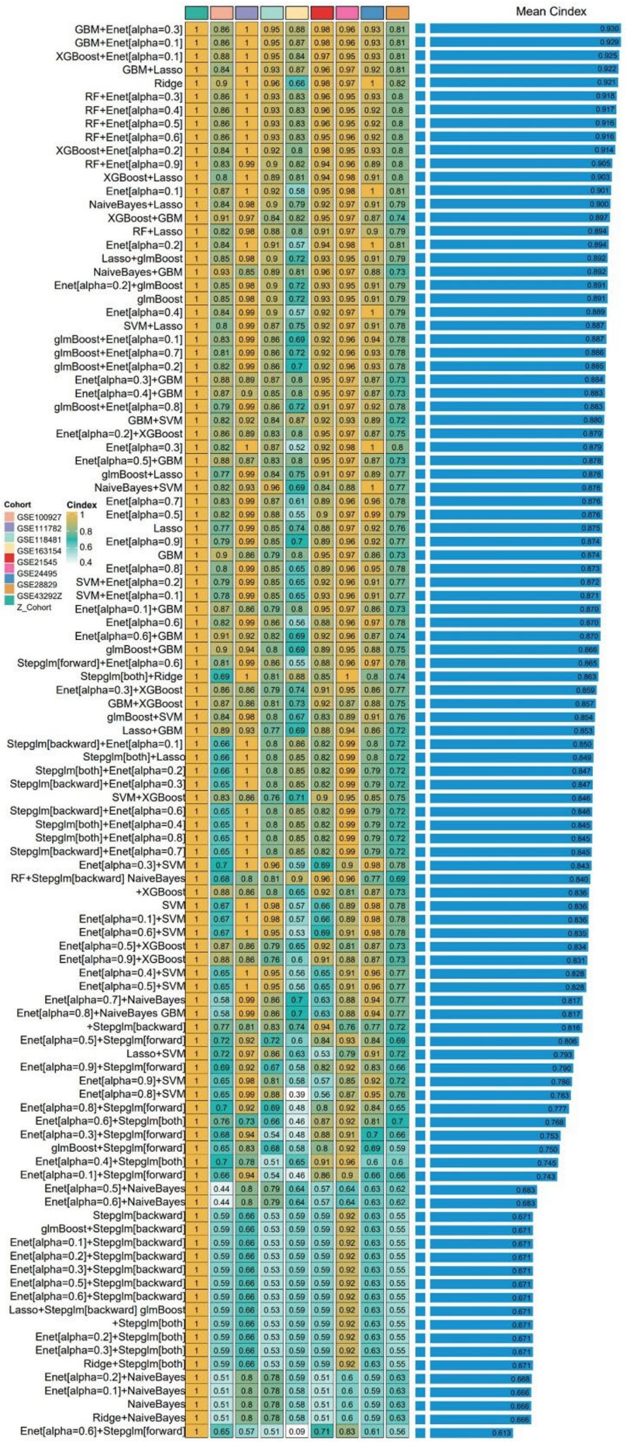

Figure S2. Comparison of 105 machine learning models across training and validation cohorts.

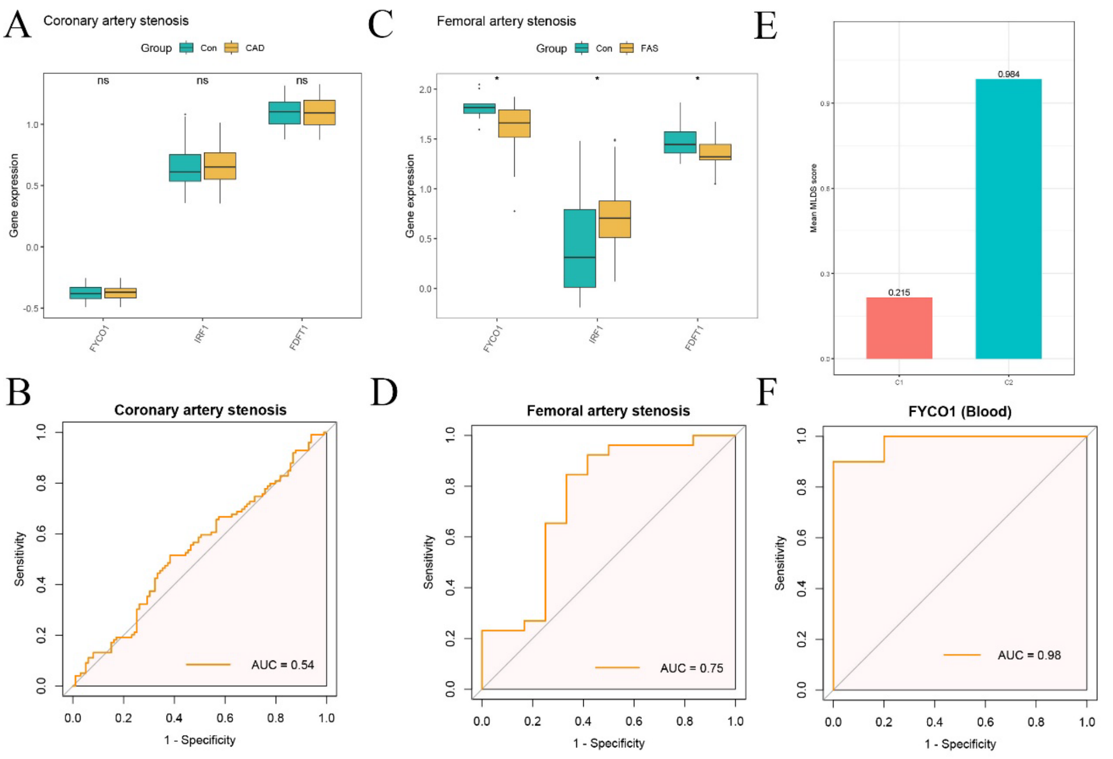

Figure S3. Evaluation of MLDS specificity across different arterial stenosis cohorts. (A-B) The MLDS model showed no significant differential expression (A) and poor diagnostic performance (B) in the coronary artery stenosis cohort. (C-D) In the femoral artery stenosis cohort, gene expression patterns were directionally consistent with those in CAS (C) and achieved moderate diagnostic accuracy (D). (E) MLDS scores were significantly higher in the C2 subtype compared with C1. (F) FYCO1 demonstrated strong diagnostic efficacy in peripheral blood samples. ns $P>0.05$; $*P<0.05$

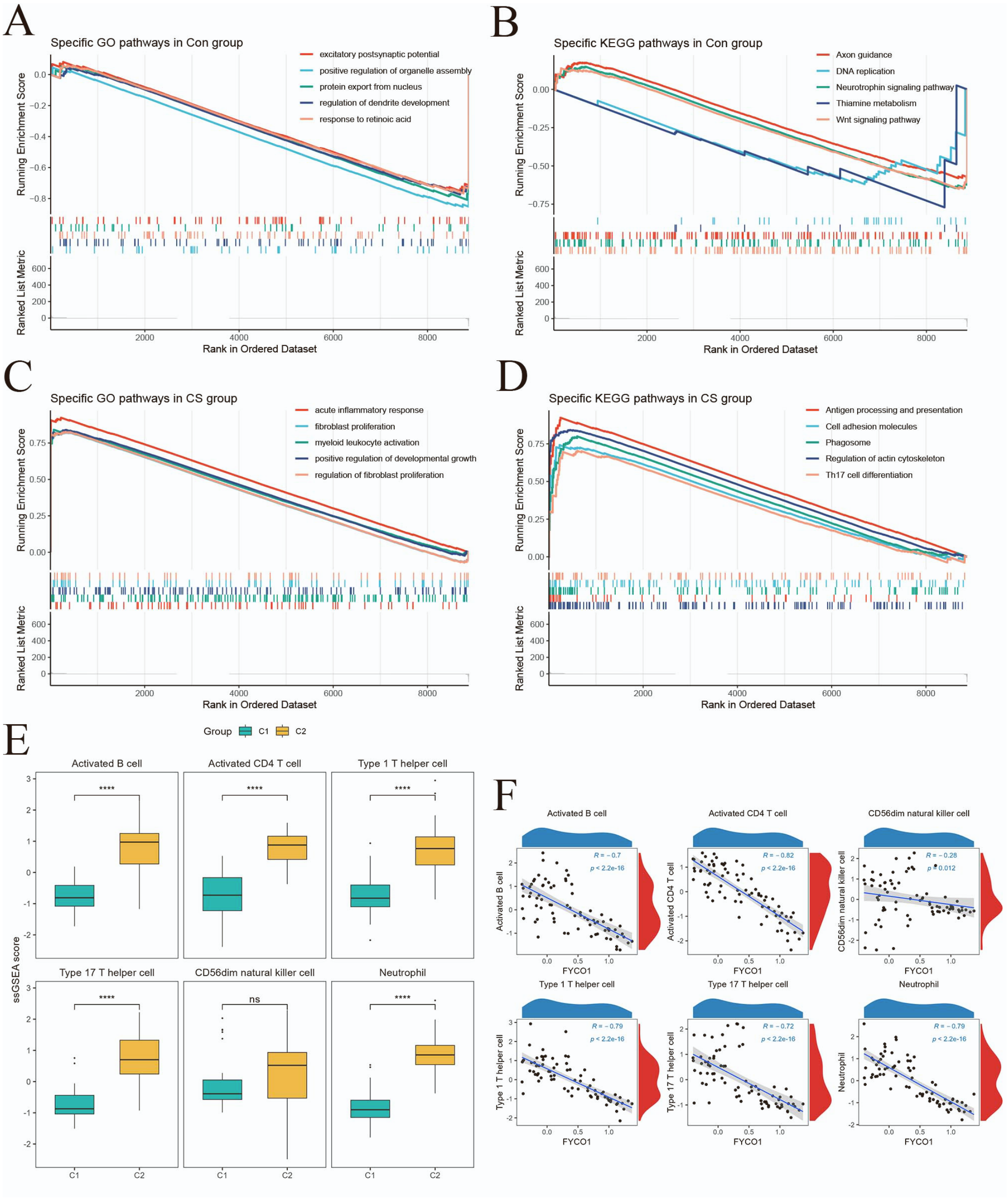

Figure S4. Functional enrichment and immune correlations of MLDS subtypes.

(A-B) GO and KEGG enrichment pathways in the low-risk group. (C-D) GO and KEGG enrichment pathways in the high-risk group. (E) Immune infiltration differences between C1 and C2 subtypes. (F) Negative correlations between FYCO1 expression and immune cell abundance across subtypes. ns P>0.05; ****P<0.0001

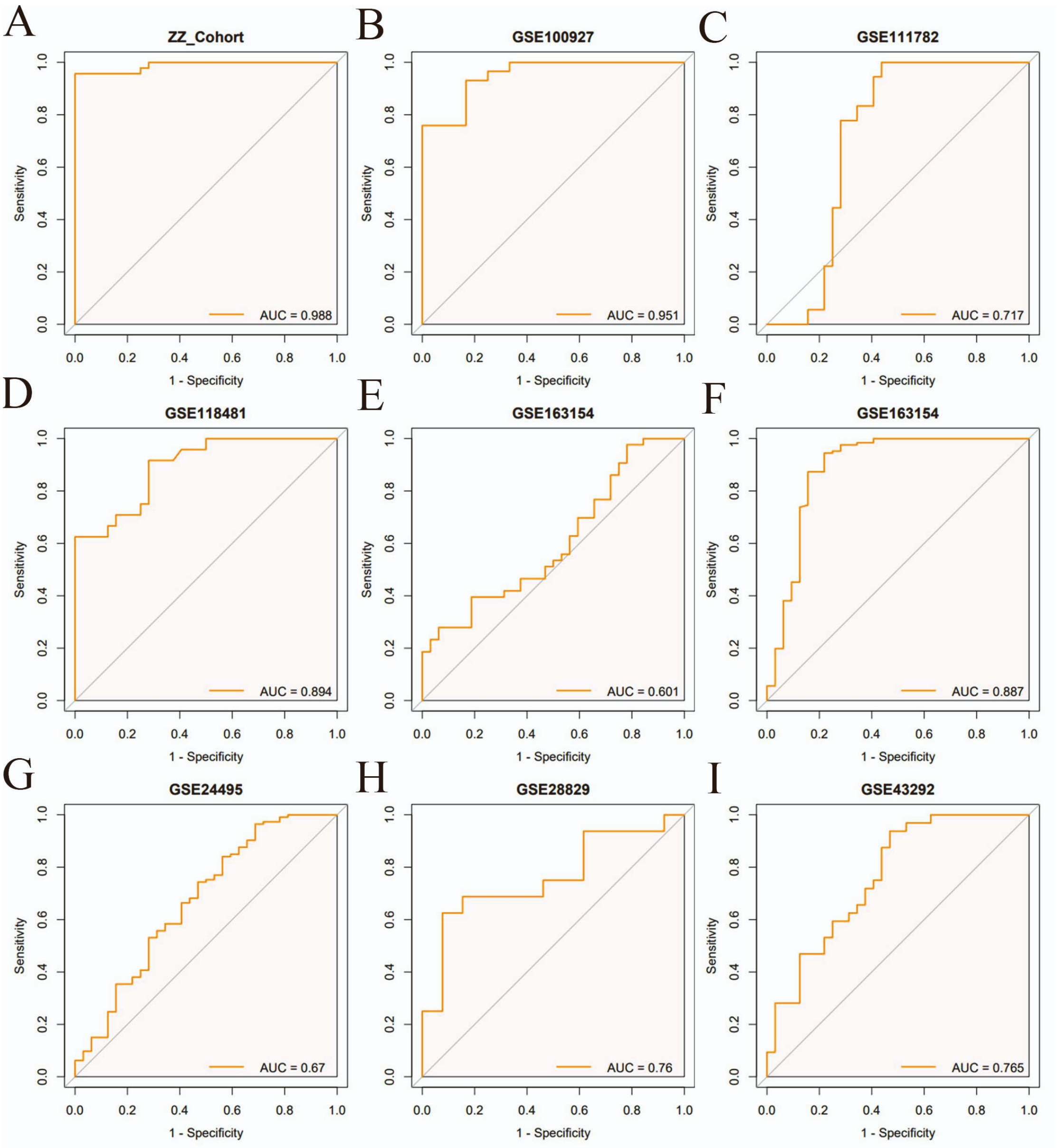

Figure S5. Diagnostic performance of FYCO1 across nine cohorts. ROC curves showing the ability of FYCO1 to distinguish CAS from controls in the training and validation datasets (A-I).

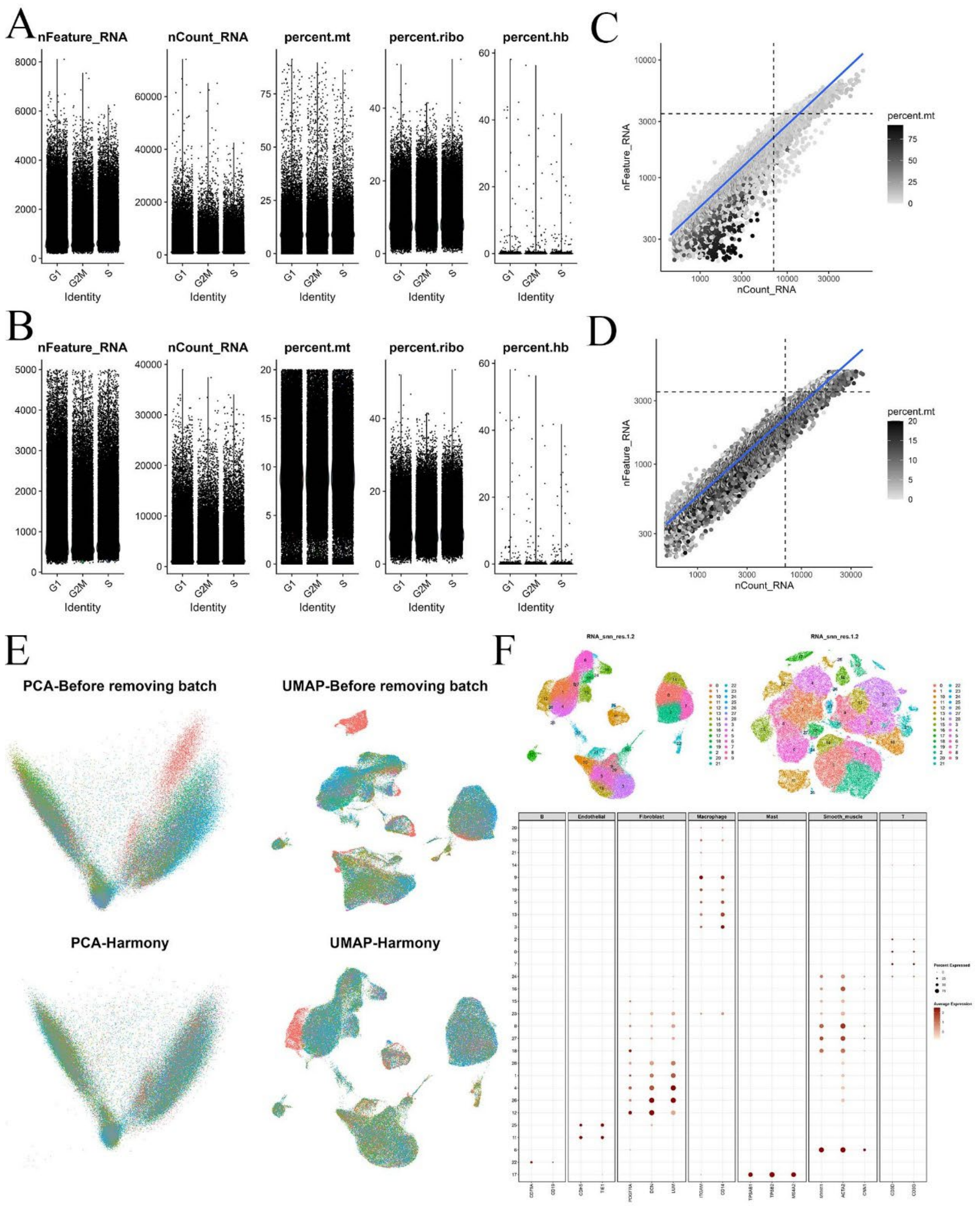

Figure S6. Quality control and cell clustering in single-cell RNA-seq.

(A-B) Quality control metrics of scRNA-seq data. (C-D) Correlation between detected genes and sequencing depth. (E) PCA and UMAP before and after batch correction. (F) UMAP visualization of major cell populations and cell-type-specific expression distribution.
