# Supplementary material for: Consensus machine learning identifies cell death gene signature for carotid artery stenosis diagnosis
Source: iScience. 2025 Dec 13;29(2):114397. doi: 10.1016/j.isci.2025.114397 (PMC12874109; doi:10.1016/j.isci.2025.114397)
Supplement: Supplementary Methods [file mmc2.pdf]

Supplementary methods

1. Consensus machine learning framework and hyperparameter tuning

To construct the consensus diagnostic signature, we implemented a unified machine-learning pipeline in R (version 4.4.2) based on ten commonly used binary classifiers: least absolute shrinkage and selection operator (LASSO), ridge regression, elastic net (Enet), stepwise logistic regression (Stepglm; backward, forward, or both directions), random forest (RF), generalized linear model boosting (glmBoost), gradient boosting machine (GBM), support vector machine (SVM), naïve Bayes, and eXtreme gradient boosting (XGBoost). All models were trained on the ZZ-Cohort, and their performance was evaluated across the remaining eight external cohorts.

Overall training scheme and cross-validation:

For each of the 105 algorithmic combinations (e.g., "GBM+Enet[α=0.3]"), the pipeline consisted of two stages:

- an optional feature-selection stage.
- a learner stage that produced predicted probabilities of CAS.

In combinations of the form "Learner+Selector" (e.g., GBM+Enet), the selector (Enet, Stepglm, RF, glmBoost, Naïve Bayes, SVM, GBM, or XGBoost) was first applied in the ZZ-Cohort to select informative genes from the 14 consensus diagnostic-related genes (CDRGs). The selected features were then used to train the learneron the ZZ-Cohort.

To avoid information leakage, all feature selection and hyperparameter tuning were performed exclusively within the ZZ-Cohort. Predicted probabilities were then generated for each of the nine cohorts, and the area under the ROC curve (AUC, denoted as "C-index") was calculated in each validation cohort. The mean AUC across the eight external cohorts was used as the primary criterion to rank the 105 models.

Hyperparameter tuning strategy by algorithm

- LASSO and Ridge (glmnet): LASSO (α = 1) and ridge regression (α = 0) were implemented using the glmnet package. For each model, the regularization parameter λ was optimized by 10-fold cross-validation via cv.glmnet (family = "binomial"), using the automatically generated logarithmic grid of λ values. The value corresponding to the minimum cross-validated deviance (lambda.min) was selected and used to refit the final model on the full ZZ-Cohort.
- Elastic net (Enet): Elastic net was also implemented with glmnet. The mixing parameter α was prespecified on a discrete grid α ∈ {0.1, 0.2, …, 0.9} and encoded in the model labels (e.g., "Enet[α=0.3]"). For each fixed α, λ was tuned by 10-fold cross-validation using cv.glmnet in the ZZ-Cohort, and lambda.min was selected. These Enet models were used either directly as learners or as feature selectors in combinations such as "GBM+Enet[α=0.3]". The final top-performing model corresponded to GBM+Enet with α = 0.3.
- Stepwise logistic regression (Stepglm): Stepwise logistic regression was fitted with base glm (family = binomial), followed by stepwise selection using step with directions "backward", "forward", or "both", as specified in the model label. The full model including all candidate genes was used as the starting model, and Akaike information criterion (AIC) was used as the stopping rule. No additional grid search was applied beyond the stepwise procedure itself.
- Random forest (RF): RF models were implemented with the ranger package, using the formula interface and probability = TRUE. We used 1,000 trees (num.trees = 1000) and a minimum node size of 4 (min.node.size = 4), which provided stable results in preliminary runs. Variable importance was assessed using permutation importance, and genes with normalized importance > 0.2 were retained as RF-selected features; when fewer than two features met this criterion, the top-ranked genes were kept. The number of trees and minimum node size were kept fixed rather than tuned over an additional grid.
- GLM boosting (glmBoost): Generalized linear model boosting was implemented via mboost::glmboost with a binomial loss (family = mboost::Binomial()). The number of boosting iterations (mstop) was selected by cross-validated risk

minimization using cvrisk with k-fold cross-validation on the ZZ-Cohort. After choosing the optimal mstop, the model was refitted and used either as a learner or as a feature selector based on its variable importance (varimp).

- Gradient boosting machine (GBM): GBM models were fitted with the gbm package using a Bernoulli loss (distribution = "bernoulli"). In the feature-selection stage (sel_gbm), we used n.trees = 3,000, interaction.depth = 3, shrinkage = 0.01, n.minobsinnode = 10, and 5-fold cross-validation (cv.folds = 5) to determine the optimal number of trees via gbm.perf. In the learner stage (learn_gbm), GBM was trained with n.trees = 8,000, interaction.depth = 3, shrinkage = 0.005, n.minobsinnode = 10, and 10-fold cross-validation (cv.folds = 10); the final number of trees (**n.trees*) was selected with gbm.perf and then used for prediction. Other GBM hyperparameters (depth, shrinkage, minimum node size) were fixed to these empirically chosen values.
- XGBoost: XGBoost models were implemented via xgboost. Data were stored in xgb.DMatrix objects, and a binary logistic loss was used (objective = "binary:logistic", eval_metric = "auc"). We used max_depth = 3, eta = 0.1, subsample = 0.9, and colsample_bytree = 0.9. The primary tuned hyperparameter was the number of boosting rounds (nrounds), which was selected by 5-fold cross-validation with early stopping (xgb.cv, nfold = 5, early_stopping_rounds = 20). The best iteration determined by cross-validation was then used to fit the final model on the full ZZ-Cohort. No additional random or grid search over (max_depth, eta, subsample, colsample_bytree) was performed.
- Naïve Bayes: Naïve Bayes was implemented using e1071::naiveBayes. Continuous predictors were standardized, non-finite values were imputed with column medians, and near-zero variance features were removed. No additional hyperparameters were tuned, as this classifier was mainly used as a simple learner or feature selector.
- Support vector machine (SVM): SVM models were fitted using e1071::svm with a radial basis function kernel (kernel = "radial") and C-classification. We used cost = 1 and gamma = 1/p (where p is the number of predictors) as standard, robust

defaults, and enabled probability estimation (probability = TRUE). Because SVM served as one component in the large model grid and overall performance was dominated by the outer consensus framework, we did not further tune C and gamma via a separate grid search.

2. Final GBM + Enet Model

- GBM Feature Selection: A gradient boosting machine (GBM) was first fitted using all candidate diagnostic genes (distribution = "bernoulli", n.trees = 3000, interaction.depth = 3, shrinkage = 0.01, n.minobsinnode = 10, cv.folds = 5). The optimal number of trees was determined by cross-validation (gbm.perf). Genes with ≥2% relative influence were selected; if fewer than two genes met this threshold, the top-ranked predictors were retained. \
- Elastic Net Model (α = 0.3): Using the GBM-selected features, an elastic net logistic regression model was trained with glmnet. The regularization parameter λ was chosen by 10-fold cross-validation, and lambda.min was used as the final penalty value. Genes with non-zero coefficients at λ_min constitute the final MLDS signature.
- External Validation: The fitted Enet model was applied to all nine cohorts, using the same GBM-selected features and standardization procedure. Predicted probabilities were evaluated using AUC (via the pROC package). The mean AUC across the eight external cohorts was used to quantify overall diagnostic performance.
